# PTK2-associated gene signature could predict the prognosis of IPF

**DOI:** 10.1186/s12931-023-02582-4

**Published:** 2023-12-06

**Authors:** Anlin Feng, Yesenia Moreno Caro, Colin Gardner, Garrett Grischo, Ying Liang, Praveen D. Wickremasinghe, Michaela Polmann, Mrinalini Kala, Timothy Marlowe, Stephen M. Black, Kenneth S. Knox, Ting Wang

**Affiliations:** 1 Center for Translational Science, and Department of Environmental Health, Florida International University, Port St. Lucie, FL 36987 USA; 2 Department of Internal Medicine, University of Arizona, Phoenix, AZ 85004 USA; 3 Herbert Wertheim College of Medicine, Florida International University, Port St. Lucie, FL 33199 USA; 4 Center for Translational Science, Florida International University, 11350 SW Village Pkwy, Port St. Lucie, FL 34987 USA

## Abstract

Idiopathic pulmonary fibrosis (IPF) is a progressive and fatal lung disease with a poor prognosis. Current/available clinical prediction tools have limited sensitivity and accuracy when evaluating clinical outcomes of IPF. Research has shown that focal adhesion kinase (FAK), produced by the protein tyrosine kinase 2 (PTK2) gene, is crucial in IPF development. FAK activation is a characteristic of lesional fibroblasts; Thus, FAK may be a valuable therapeutic target or prognostic biomarker for IPF. This study aimed to create a gene signature based on PTK2-associated genes and microarray data from blood cells to predict disease prognosis in patients with IPF. PTK2 levels were found to be higher in lung tissues of IPF patients compared to healthy controls, and PTK2 inhibitor Defactinib was found to reduce TGFβ-induced FAK activation and increase α-smooth muscle actin. Although the blood PTK2 levels were higher in IPF patients, blood PTK level alone could not predict IPF prognosis. From 196 PTK2-associated genes, 11 genes were prioritized to create a gene signature (PTK2 molecular signature) and a risk score system using univariate and multivariate Cox regression analysis. Patients were divided into high-risk and low-risk groups using PTK2 molecular signature. Patients in the high-risk group experienced decreased survival rates compared to patients in the low-risk group across all discovery and validation cohorts. Further functional enrichment and immune cell proportion analyses revealed that the PTK2 molecular signature strongly reflected the activation levels of immune pathways and immune cells. These findings suggested that PTK2 is a molecular target of IPF and the PTK2 molecular signature is an effective IPF prognostic biomarker.

**Supplementary Information:**

The online version contains supplementary material available at 10.1186/s12931-023-02582-4.

## Introduction

Idiopathic pulmonary fibrosis is the most prevalent and the most fatal form of idiopathic interstitial pneumonias, with an average survival rate of 2.5 to 3.5 years [1]. Although the precise cause of IPF is not yet understood, it is believed to result from repetitive alveolar damage coupled with dysfunctional alveolar wound-healing molecular mechanisms [2], an increase in myofibroblasts, and abnormalities in macrophages and fibrocytes [3]. Identifying effective IPF biomarkers could help clinicians more accurately predict outcomes, monitor disease progression, and guide treatment decisions for IPF patients. Additionally, new markers could also provide insights into the underlying mechanisms of the disease and could potentially lead to the development of new therapies.

Currently, forced vital capacity (FVC) is the most commonly used prognostic marker for IPF [4]. FVC may not always accurately reflect disease severity, particularly in early stage patients or those with preserved lung volumes. FVC is often affected by factors such as age, gender, and height and may not be sensitive enough to detect subtle changes in disease progression. In addition to FVC, several other prognostic tools are used for IPF, including high-resolution computed tomography (HRCT) [5], gender, age, lung physiology (GAP) index [6], the composite physiologic index (CPI) [7], and blood biomarkers [8]. These tools provide additional information and may be used to predict mortality and disease progression, but accuracy is limited. Therefore, the development of new and improved prognostic markers for IPF is an important area of research.

Transforming growth factor (TGF)-β activation has been implicated in both IPF and airway remodeling [9]. It is considered a central pro-fibrotic cytokine in IPF [9]. TGF-β1 promotes myofibroblast differentiation and enhances fibrotic responses in the lung [10]. Myofibroblasts are recognized as the primary cell type responsible for increasing production of extracellular matrix (ECM) proteins and activating focal adhesion kinase (FAK) [11]. FAK, an integrin-associated cytoplasmic tyrosine kinase encoded by the protein tyrosine kinase 2 (PTK2) gene, plays a critical role in organ fibrosis and the development of fibrotic disorders [12, 13]. As such, it is a potential target for anti-fibrotic therapy in IPF. Several studies of mouse models have shown that FAK inhibitors may block TGF-β1-induced myofibroblast differentiation, reduce ECM production, and alleviate pulmonary fibrosis [14–16].

Blood biomarkers have been shown to correlate with the progression and development of IPF [8]. However, these biomarkers remain experimental and are not widely used in clinical trials. Non-invasive biomarkers that reveal specific pathways and gene sets across blood samples from IPF patients could be valuable tools for determining disease stages, predicting clinical outcomes, and assisting in selecting personalized treatments. Despite TGF-β1-ECM-FAK being recognized as key proteins in IPF and FAK inhibitors being used in several IPF animal model studies [14, 15], no research has focused on a FAK-based gene signature for IPF prognosis.

In this study, we confirmed that FAK is upregulated in IPF lung tissue and therefore FAK inhibition can be used as a therapeutic strategy against TGF-β1-induced ECM remodeling. Importantly we identified a PTK2-associated gene signature by analyzing our discovery cohort and the STRING database using univariate and multivariate COX regression analyses. We included 11 genes to create a risk score prognostic system. We verified this gene signature in discovery and validation cohorts, demonstrating that this risk score system is an independent and robust prognostic gene signature for IPF.

## Methods

### Acquisition of datasets

To determine PTK2 expression levels in IPF patients and healthy controls, we conducted a systematic search of the GEO database using the keywords “idiopathic pulmonary disease,” “lung tissues,” and “microarray.” The criteria for inclusion included: investigations that had IPF patients and healthy individuals as healthy control, microarray datasets, and studies that utilized samples from lung tissues. On the other hand, the criteria for exclusion included: studies based on animal research, application of RNA-sequencing datasets, lack of a healthy control group, and studies that provided data derived from non-lung tissue samples. Many studies were excluded due to presence of animal research, RNA-sequencing data, and non-lung tissue samples, or the absence of healthy controls. We included three datasets in this study (Table 1), from which we downloaded and normalized microarray data. The transcripts per kilobase million (TPM) normalization method was used for gene expression normalization in this study. Three microarray datasets [17–19], comprising 98 lung tissue samples in total, were included in this study. These IPF lung tissue samples were collected from diagnostic surgical biopsy or transplantation involving IPF patients. On the other hand, normal lung tissues were gathered from surrounding tissues during lung cancer resections or standard lung volume reduction from healthy donors. We then extracted sample count, mean, and standard deviation values for both healthy controls and IPF patients from the microarray data. A random-effects model meta-analysis was performed using the R package “meta” (version 6.2-1).

**Table 1:** Datasets Summary

	Type	Sample Count (IPF/Healthy Control)	Platform
GSE24206	Lung tissue	17 (11/6)	Affymetrix Human Genome U133 Plus 2.0 Array
GSE110147	Lung tissue	33 (22/11)	Affymetrix Human Gene 1.0 ST Array
GSE53845	Lung tissue	48 (40/8)	Agilent-014850 Whole Human Genome Microarray

Additionally, we carried out a systematic search in the GEO database for datasets containing clinical outcomes for IPF patients. The keywords used were “idiopathic pulmonary disease,” “clinical outcome,” “microarray,” “survival,” and “blood.” The criteria for inclusion included: microarray datasets, studies that contained clinical outcomes, and those that used blood samples. On the other hand, our exclusion criteria included: animal-based research studies, use of RNA-sequencing datasets, studies without clinical outcome data, and studies that provided data derived from non-blood samples. Three datasets were included in this study (Table 2), and microarray data were downloaded and normalized accordingly. The three microarray datasets [20–22], including 172 peripheral blood samples from IPF patients, were utilized to generate and validate a prognostic gene signature. These IPF patients were tracked from the point of blood collection until death, transplant, or final follow-up. Thus, clinical outcomes have been recorded, including time to such outcomes. GSE27957 served as the discovery cohort, while GSE28042 and GSE93606 functioned as validation cohorts.

**Table 2:** Demographic summary

	GSE27957		GSE93606		GSE28042	
	Count	Percentage (%)	Count	Percentage (%)	Count	Percentage (%)
Sex						
Male	38	84.44	33	64.71	44	57.89
Female	7	15.56	18	35.29	32	42.11
Clinical outcome						
Survivors	32	71.11	20	39.22	25	32.89
Non-survivors	13	28.89	31	60.78	51	67.11
Age (years)						
< = 65	23	51.11	24	47.06	29	38.16
> 65	22	48.89	27	52.94	47	61.84

### Identification of PTK2-related genes

We obtained PTK2-related genes and protein–protein interaction (PPI) networks from the STRING database (https://string-db.org/), using the following settings: inclusion of all active interaction sources, high confidence interaction score (0.7), and a maximum of 500 interactors. We are using "FAK" as the protein name input and "Homo sapiens" as the organisms input. Only the first shell containing proteins that directly interact with FAK was included in this study. Tab-separated value files were downloaded for further analysis, and KEGG analysis was also conducted within the database.

### Kaplan–Meier analysis for the PTK2 single-gene model

The patients in these datasets were divided into PTK2 high and low groups using the median PTK gene expression value as the cutoff. The Kaplan–Meier method was used to estimate the survival curves of IPF patients, which was carried out using R packages “survminer” (version 0.4.9) and “survival” (version 3.5-5). The groups were compared via the log-rank test.

### Construction of risk score prognostic model

Univariate and multivariate Cox regression analyses, employing the same R packages as the Kaplan–Meier method, were conducted to assess the prognostic impact and create a gene signature model for prediction. To analyze the prognostic roles of the PTK2-related genes, we conducted univariate Cox regression analysis in the discovery cohort. IPF patients without follow-up data were excluded from this analysis. Genes with a log-rank P value < 0.05 were considered significant and identified as IPF survival-associated genes (Table 3). Subsequently, a multivariate Cox regression analysis was performed to select these IPF survival-associated genes for the development of a risk-scoring system. Genes with a log-rank P value < 0.05 were included in this system (Table 4). The formula for the risk-scoring system is presented as follows:

$$risk \,score=\sum \limits_{i=1}^{n}\left({e}_{i}*{\beta }_{i}\right)$$

n: The number of genes included in PTK2-related gene signature; e_*i*_: Standardized gene expression of the *i*th gene; β_*i*_: Regression coefficient of the *i*th gene.

**Table 3:** 44 gene Signature Summary

Gene Name	Coefficient	Hazard ratio	P_value
ACTA1	5.100	160.000	0.012
ACTN1	1.300	3.700	0.008
ARHGEF6	− 2.500	0.082	0.005
ARHGEF7	− 5.600	0.004	0.013
ASAP1	1.900	6.700	0.013
BMX	2.100	8.500	0.006
CASP6	− 1.700	0.190	0.016
CASP7	− 2.200	0.110	0.001
CAST	− 1.500	0.210	0.010
EDIL3	1.200	3.500	0.007
EZR	− 2.400	0.090	0.003
FCER1A	− 0.780	0.460	0.009
FGR	2.800	17.000	0.017
FLT1	4.100	62.000	0.014
FYN	− 2.200	0.110	0.008
HCK	1.400	4.100	0.040
HSP90AA1	− 2.000	0.140	0.026
ITGA4	− 2.700	0.066	0.002
ITGA5	1.900	6.900	0.023
ITGB7	− 1.700	0.180	0.008
LCK	− 1.600	0.200	0.001
MAPK1	3.400	29.000	0.016
MAPK8IP3	5.700	310.000	0.007
MEF2C	− 2.500	0.083	0.012
NCK1	− 2.400	0.093	0.001
NOLC1	− 2.500	0.081	0.006
PIK3CA	− 2.200	0.110	0.016
PIK3R1	− 2.100	0.120	0.001
PIK3R3	− 3.500	0.031	0.002
PLCG1	− 3.000	0.050	0.000
PRKACB	− 1.600	0.210	0.001
PTEN	2.000	7.200	0.012
PTPN13	− 4.200	0.015	0.002
PVRL1	4.000	54.000	0.015
RASA1	− 1.700	0.190	0.049
SH3GL1	5.900	370.000	0.005
SIRPA	1.100	3.000	0.026
SOCS3	1.900	6.400	0.010
SPTAN1	− 2.700	0.065	0.002
STAT3	1.800	6.300	0.017
SYK	2.200	8.800	0.012
TLN1	1.800	5.800	0.015
UTRN	− 2.100	0.120	0.008
WAS	2.300	10.000	0.032

**Table 4:** 11 gene signature summary

Gene Name	Coefficient	P_value
ACTN1	− 17.5	0.007
ASAP1	18.9	0.004
BMX	16.3	0.007
EDIL3	4.94	0.037
FGR	29	0.022
HCK	− 11.7	0.028
ITGA5	27.4	0.026
MAPK8IP3	22.3	0.049
PTEN	13.9	0.013
SIRPA	− 23.9	0.003
SOCS3	15.4	0.009

### Validation of the risk score prognostic model

Time-dependent ROC curve analysis was performed using the R package “timeROC” (version 0.4). The violin plots were generated by the R package “ggplot2” (version 3.4.0). All analyses were two-tailed, and a p-value < 0.05 was considered statistically significant.

The patients in these datasets were divided into high-risk and low-risk groups using the median risk score as a cutoff. We identified differentially expressed genes (DEGs) between the high-risk and low-risk patients with a |fold change (FC)|> 1.2 and false discovery rate (FDR) < 0.05 as cutoffs, utilizing the R package “limma” (version 3.54.2). For functional enrichment analysis, we performed GSVA and GSEA analyses using the R packages “GSVA” (version 1.46.0) and “GSEA-MSigDB/GSEA_R” (version 1.2). Heatmaps were created with the R package “gplots” (version 3.1.3), while gene correlations and corresponding heatmaps were analyzed and generated using the R packages “Hmisc” (version 5.0-1), “ggplot2” (version 3.4.0), and “corrplot” (version 0.92).

### Estimation of immune cell proportions

As immune cells and the immune system play crucial roles in IPF progression, we assessed the proportions of immune cells in the blood and compared these between the high-risk and low-risk groups. The Cibersort algorithm (R script, version 1.04) was employed to estimate immune cell proportions in the discovery cohort.

### Cell culture and western blot

Human lung fibroblast cells (IMR90) were obtained from ATCC (CCL-186) and maintained in DMEM medium supplemented with 10% fetal bovine serum (FBS) and 1% penicillin/streptomycin at 37 °C with 5% CO_2_ atmosphere. At 70% cell confluency, we serum-starved the IMR90 for 24 h. A commercially available FAK inhibitor, Defactinib (2 μM), was utilized to treat TGF-β (2 ng/ml) matured IMR90 at passage 5 in vitro.

DHLF-IPF, diseased human adult lung fibroblasts, were obtained from Lonza (CC-7231) and maintained in FBM-2 fibroblast growth medium at 37 °C with a 5% CO_2_ atmosphere. When the cell confluency reached 70%, we treated the DHLF-IPF cells with Defactinib (2 μM) at passage 5 in vitro.

The evaluation of FAK phosphorylation and typical fibrosis markers (pY397-FAK, FAK, and alpha smooth muscle actin (α-SMA)) was conducted through western blot analysis. A series of primary antibodies including anti-pY397-FAK (1:1000; D20B1, Cell Signaling), anti-FAK (1:1000; 3285T, Cell Signaling), anti-⍺-SMA (1:1000; A2547, Sigma) and anti-β-actin (1:1000; A1978, Sigma) were used in this study.

### Statistical analyses

GraphPad Prism software (GraphPad Software, San Diego, CA) was used to perform all statistical calculations for experiments other than transcriptomic analysis. The means ± SDs were calculated, and significance was determined by either the unpaired t-test or ANOVA. For ANOVA, Tukey’s multiple comparisons test was utilized. A p-value < 0.05 was considered significant.

## Results

### FAK is upregulated in IPF lungs

**Fig. 1:**
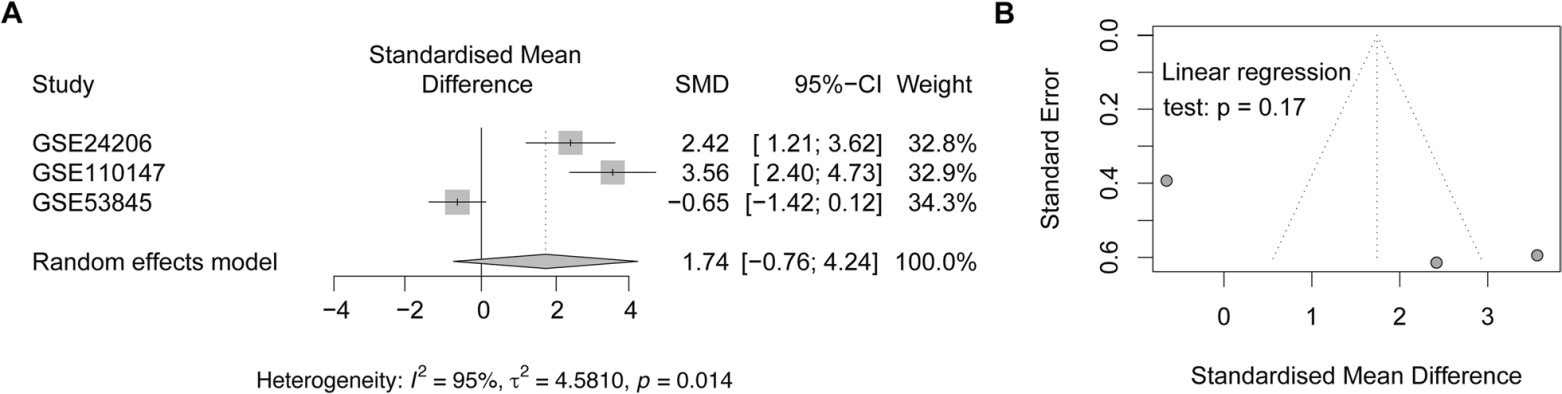
Meta analysis of gene expression of PTK2 in IPF patients (**A**–**B**). Gene expression of FAK/PTK2 was higher in lung tissue samples of individuals with IPF when compared to healthy controls (**A**)

The confirmation of PTK2 gene expression up-regulation in IPF has yet to be established. We decided to employ a meta-analysis to examine PTK2 gene expression in IPF lung tissues using the GEO database, including three transcriptomic datasets in our research (Table 1). Forest plots with a random effect model revealed higher PTK2 gene expression levels in IPF compared to healthy controls (heterogeneity p-value: 0.014, Fig. 1A). The funnel plot depicted in Fig. 1B demonstrates an absence of bias in the included studies. Based on all three transcriptomic studies, we have confirmed an increase in FAK/PTK2 expression in lung tissues from IPF patients.

### FAK inhibitor Defactinib can attenuate TGF-β induced fibrosis in vitro

Next, we examined the feasibility of inhibiting FAK to treat IPF. Defactinib, a highly selective FAK inhibitor was used to treat TGF-β (2 ng/ml) challenged lung fibroblast cells (IMR90) and DHLF-IPF cells in vitro. We assessed FAK phosphorylation (pY397-FAK) and common fibrosis marker alpha smooth muscle actin (α-SMA) via western blot (Additional file 2: Fig. S1A, B, Additional file 3: Fig. S2A, B, Additional files 4, 5, 6, 7). The α-SMA is commonly utilized as an indicator for identifying subsets of fibroblasts that drive the progression of fibrosis. Defactinib diminished both pY397-FAK and fibrotic marker α-SMA in TGF-β activated fibrotic IMR90 and DHLF-IPF cells. Meanwhile, we observed that Defactinib could restore the TGF-β remodeled cellular morphology of IMR9 and DHLF-IPFAQ5 cells (Additional file 2: Fig. S1C, Additional file 3: Fig. S2C). These data confirmed that FAK is a viable therapeutic target for IPF in this in vitro model of fibrosis.

### Blood PTK2 has limited diagnostic power

**Fig. 2:**
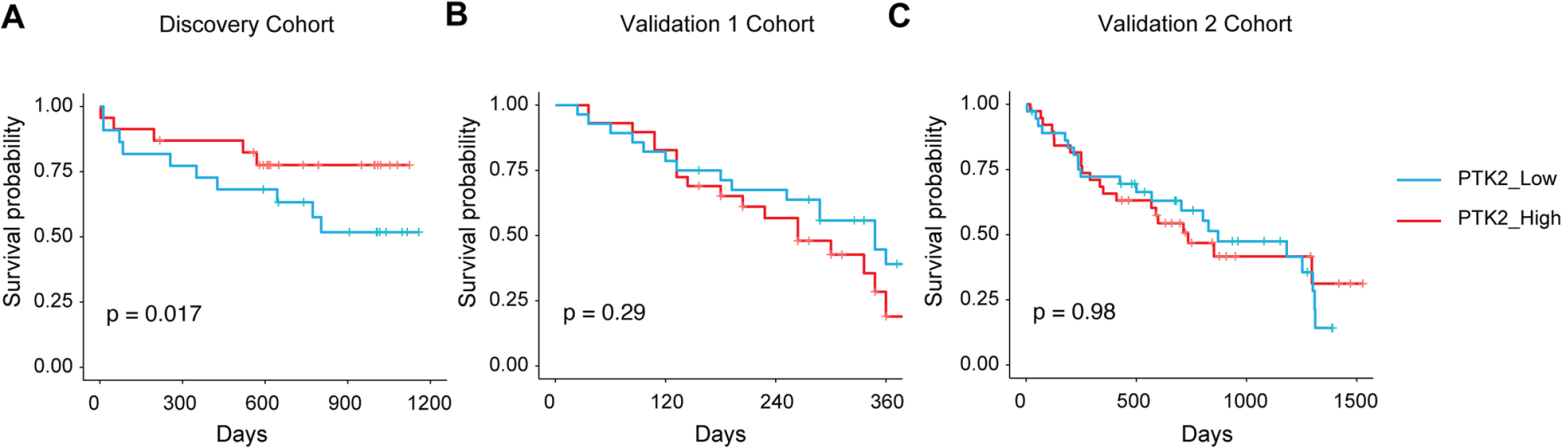
Kaplan Meier curve for single gene PTK2 in IPF. High PTK2 gene expression in blood samples was not strongly associated with prognosis of IPF (**A**–**C**)

We hereby confirm that the PTK2 gene, which encodes for FAK, is a potential biomarker for IPF. In this study, we analyzed three GEO datasets comprising IPF patients with clinical outcomes and blood samples. GSE27957 was used as the discovery cohort, while GSE28042 and GSE93606 were used as validation cohorts. Table 2 presents a summary of the demographic information for these datasets. Although FAK is increased in IPF tissues, our data does not suggest that the PTK2 gene alone can predict the prognosis of IPF patients. We observed that increased expression levels of the PTK2 gene in peripheral blood mononuclear cells (PBMCs) were associated with improved survival in IPF patients in the discovery cohort only, with no significant correlations found in the validation cohorts (Fig. 2A–C). The differences in clinical outcomes for PTK2 in IPF patients are complex, and disease processes often involve changes in the expression patterns of groups of genes with similar biological functions or strong correlations. Therefore, a significant alteration in these gene sets is more biologically reliable and interpretable than a change in a single gene such as PTK2. Consequently, we have initiated the development of a gene signature based on PTK2-related genes to better predict the prognosis of IPF patients.

### A FAK-associated gene signature model is established

**Fig. 3:**
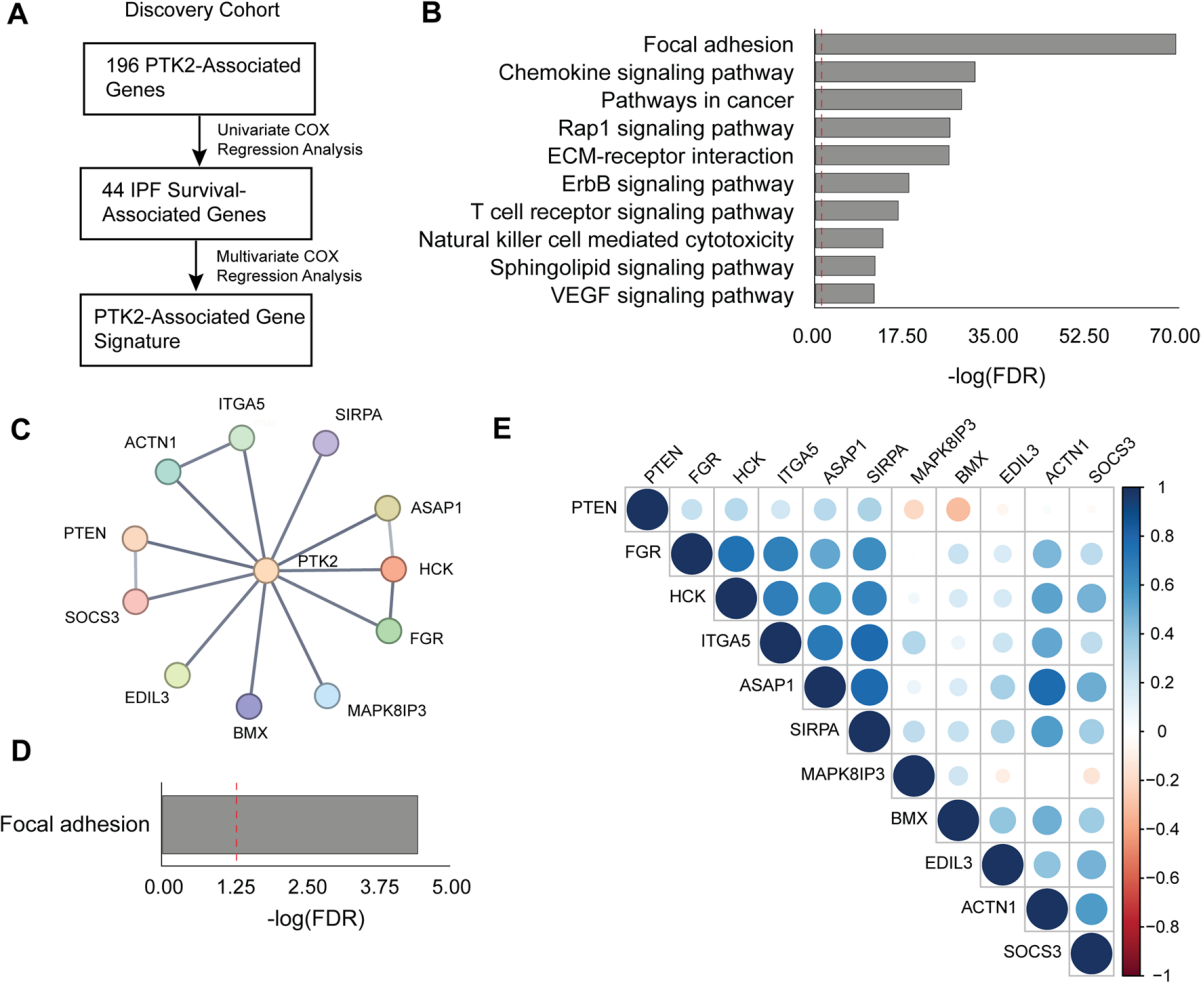
A gene signature associated with PTK2 was generated to predict the prognosis of IPF. In the discovery cohort, a pipeline illustrated in flowchart (**A**) was used to generate a gene signature associated with PTK2, followed by KEGG analysis (**B**) to identify all pathways enriched among PTK2-associated genes. The 11 genes within the PTK2-associated gene signature were then subjected to a PPI network analysis (**C**), additional KEGG analysis (**D**), and correlation heatmap analysis (**E**)

The overall study workflow is depicted in Fig. 3A. PTK2-related genes were acquired from the STRING database with the search parameters including all active interaction sources (see Additional file 1: Table S1), the highest confidence interaction scores, and the maximum number of interactors. A total of 196 genes were identified as PTK2-associated in the STRING network, yielding a significant protein–protein interaction (PPI) enrichment score (p-value for enrichment < 1.0e−16). Through Kyoto Encyclopedia of Genes and Genomes (KEGG) pathway analysis, VEGF signaling pathways, ECM–receptor interaction, and focal adhesion pathways were found to be enriched among these genes (Fig. 3B).

Subsequently, we aimed to identify IPF survival-related genes from the 196 genes by performing univariate Cox regression analysis, a method commonly employed in clinical research to examine the relationship between patient survival time and a single predictor variable. Of the 196 genes, 44 were found to be associated with IPF prognosis (Table 3). Multivariate Cox regression analysis was then used to create a gene signature, with 11 genes demonstrating significant log-rank test p-values being included in the final PTK2-associated gene signature (Table 4). These genes exhibited strong interactions within the PPI network (Fig. 3C). Notably, only the FAK-associated pathway of focal adhesion was enriched among these genes (Fig. 3D). Interestingly, the correlation matrix (Fig. 3E) revealed a strong positive correlation between a group of several genes, including FGR, HCK, ITGA5, ASAP1, and SIRPA.

### PTK2-associated gene signature is used to predict IFP survival in both discovery and validation cohorts

**Fig. 4:**
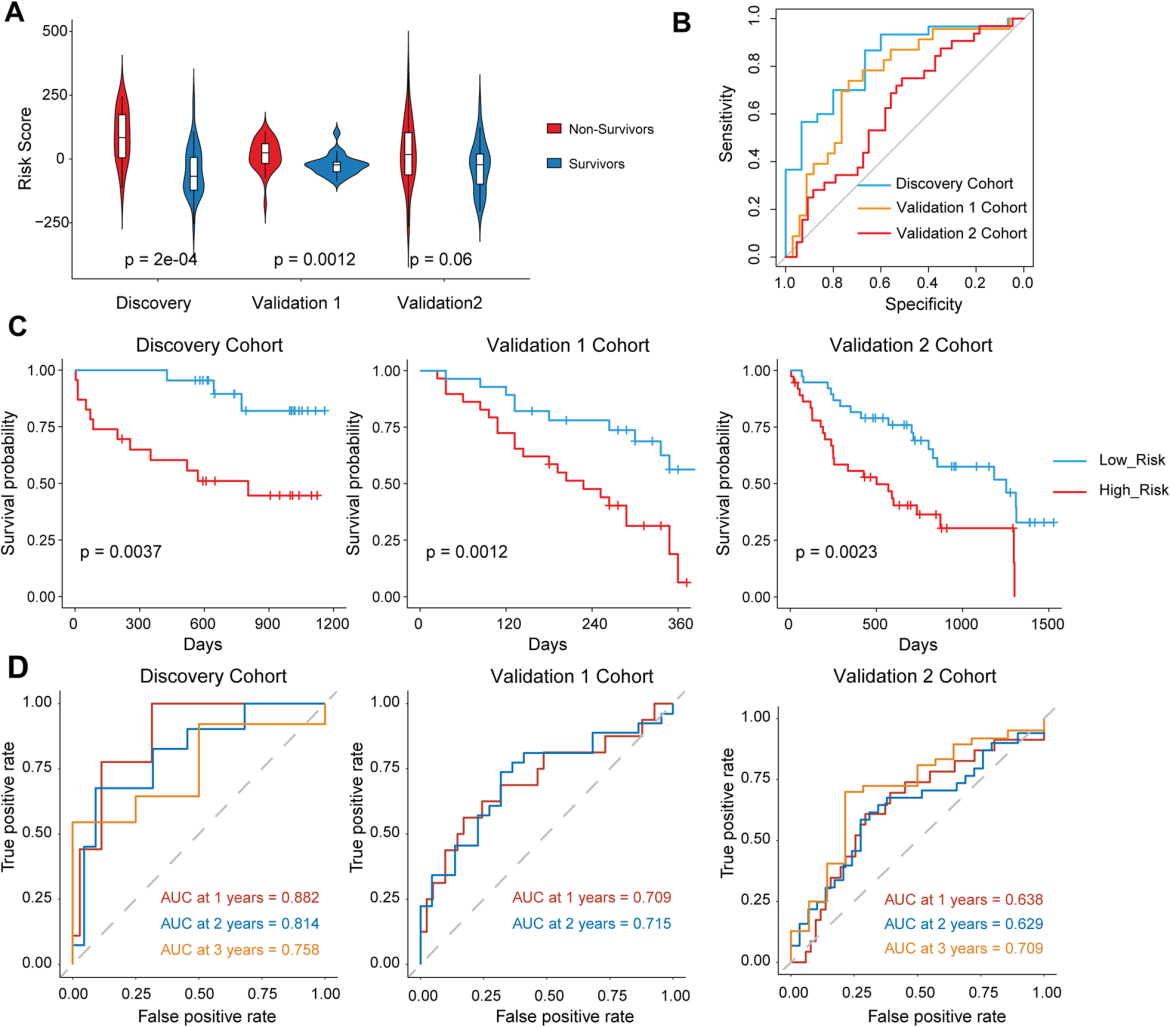
Validation of PTK2-GS in discovery and validation cohorts. Violin plot (**A**) and ROC curve (**B**) of the PTK2-GS in IPF non-survivors and survivors. Kaplan Meier Curve (**C**) and Survival ROC curve for 1, 2 and 3 years of the PTK2-GS in IPF High-Risk and Low-Risk patients

We used the expression levels and Cox coefficients of the 11 genes to calculate risk scores for individual IPF patients. First, we compared risk scores between non-survivors and survivors. Interestingly, our gene signature was able to distinguish non-survivors from survivors in both the discovery and validation 1 cohorts (Fig. 4A), which was further validated by the receiver operating characteristic (ROC) curve (Fig. 4B).

To assess the reproducibility of our risk score system, we analyzed 11-gene risk profiles in these independent IPF cohorts. Using the median value of the risk score as a cutoff, patients were divided into high-risk and low-risk groups, and Kaplan–Meier curves were performed to evaluate our risk score system's performance. Remarkably, the 11-gene risk score system significantly predicted mortality in the discovery, validation 1, and validation 2 cohorts (Fig. 4C). We also conducted ROC curves to evaluate the risk score at 1-, 3-, and 5-year overall survival. The discovery and validation 1 cohorts exhibited a relatively better performance in separating the two groups (Fig. 4D). These results reinforced the potential of PTK2-associated genes in predicting IPF patient prognosis and suggested an overlapping clinical outcome-related gene signature between PTK2 and IPF.

### Immune cell activation is found in high-risk patients

**Fig. 5:**
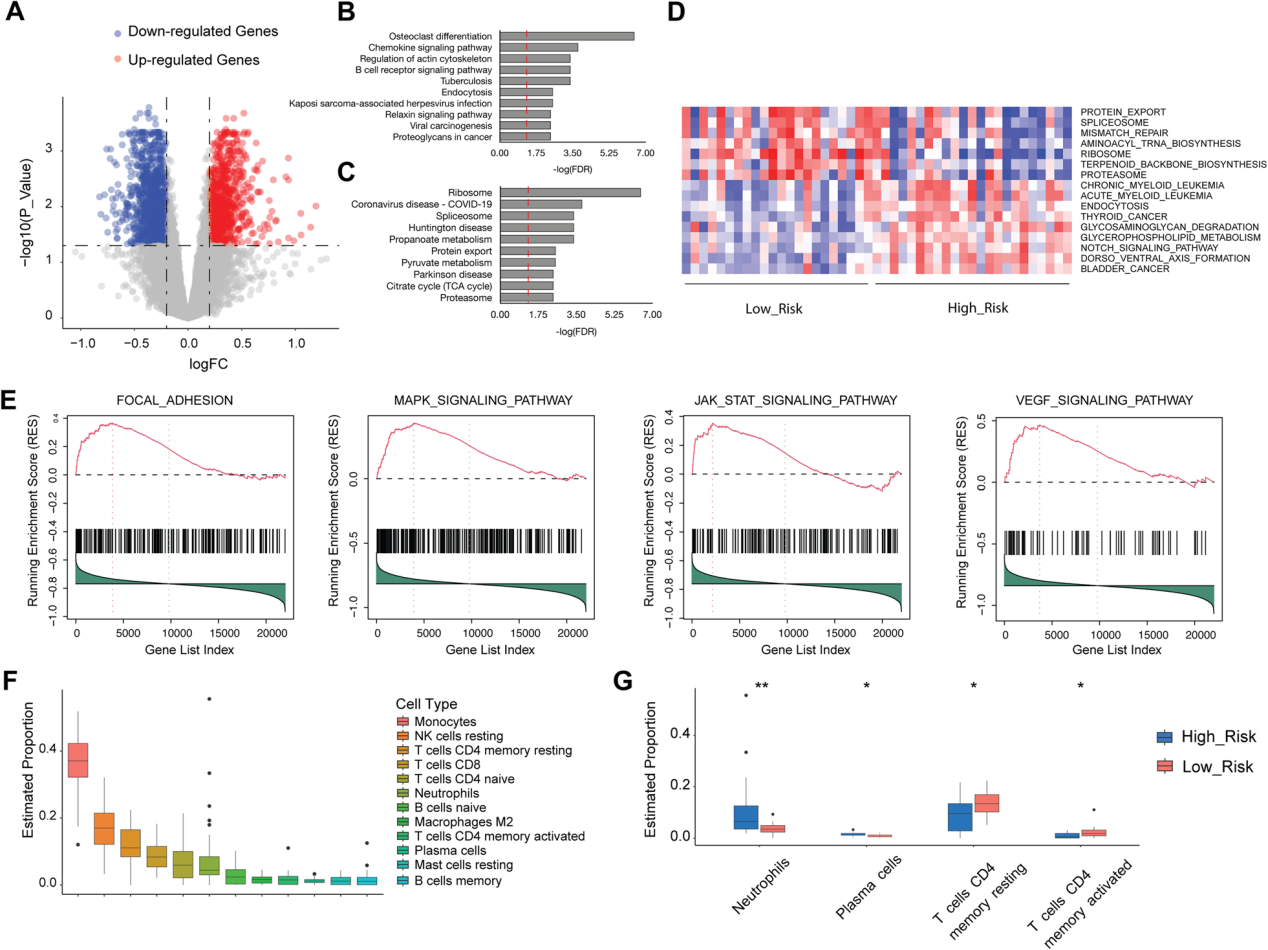
Alternation of gene expression pattern between IPF High-Risk and Low-Risk patients. Differentially expressed genes between the two groups were depicted in a volcano plot (**A**). KEGG analysis was conducted on the up-regulated (**B**) and down-regulated (**C**) genes. In the discovery cohort, gene set variation analysis (**D**) and gene set enrichment analysis (**E**) were performed to discover pathways enriched in High-Risk patients. Furthermore, Cibersort was used to estimate the immune cell proportions (**F**), and changes in immune cell proportions between High-Risk and Low-Risk patients were presented in **G**

To identify alterations in enriched pathways between high-risk and low-risk groups in the discovery phase, we analyzed differentially expressed genes (DEGs) between the two groups. A total of 577 up-regulated and 685 down-regulated genes, depicted in the volcano plot (Fig. 5A), were identified in the high-risk group. Up-regulated genes were primarily enriched in immunity or cancer-related pathways (Fig. 5B), while down-regulated genes were enriched in mRNA translation or neurodegenerative disease-related pathways (Fig. 5C). To investigate pathway activity changes in the high-risk group, we conducted gene set variation analysis (GSVA), a framework that transforms gene expression profiles into pathway expression patterns. A total of 16 significant pathways (Fig. 5D) were detected in the high-risk group, with several immunity or cancer-related pathways reappearing in the GSVA heatmap. Gene set enrichment analysis (GSEA) results, including a list of KEGG pathways, also confirmed the enrichment of focal adhesion, MAPK, JAK-STAT, and VEGF pathways in the high-risk group (Fig. 5E).

Since immune cell activation plays a crucial role in the development and progression of IPF [23–27], we analyzed estimated proportions of immune cells in these two groups to evaluate immune cell regulation in the high-risk group. We employed Cibersort, a powerful tool for assessing percentages of immune cell type in blood based on gene expression profiles from RNA-sequencing data. The estimated proportions of immune cells showed that monocytes, natural killer cells, neutrophils, and T and B cells constituted the largest portions in total blood samples from the discovery cohort (Fig. 5F). Compared to the low-risk group, the high-risk group exhibited increased percentages of neutrophils and plasma cells, along with decreased percentages of activated or resting CD4+ T cells (Fig. 5G).

## Discussion

IPF is a progressive, chronic, and fatal fibrotic lung disease. Although considered rare, its occurrence is nearly as frequent as brain and stomach cancers [28, 29]. IPF prognosis remains poor, partly due to the absence of reliable prognostic biomarkers to guide personalized treatment. The lack of effective biomarkers to predict IPF development and progression makes it challenging to determine whether patients should receive novel therapies or undergo lung transplantation. Recent evidence suggests that gene signatures in blood samples may be crucial and efficient tools for predicting IPF prognosis and development [30, 31]. As a result, establishing a risk score system based on a gene signature for IPF is crucial for predicting patient outcomes.

In this study, we identified and analyzed an 11-gene signature based on PTK2-associated genes and IPF-survival-related genes in peripheral blood from 177 IPF patients across three independent, transplant-free cohorts. The upregulation of PTK2 expression in IPF was determined through a meta-analysis. We discovered a total of 196 PTK2-associated genes in the STRING database. Out of these 196 genes, 44 were identified as survival-related genes in IPF patients using univariate COX regression analysis. Subsequently, we developed an 11-gene signature and corresponding risk score system through multivariate COX regression analysis. By employing our PTK2-associated gene signature-generated risk score system, we derived risk profiles that differentiated two IPF patient groups with significantly different clinical outcomes in all three cohorts. The findings of this study may aid in identifying high-risk patients and implementing personalized treatment in the future.

FAK is a component of Focal Adhesions (FAs), which is a complex structure formed at the cell's plasma membrane during interaction with the extracellular matrix via integrins. Acting as bridges to carry signals from outside to the cellular cytoskeleton, FAs facilitate mechanotransduction in endothelial cells. These structures, coupled with integrins and mechanotransduction pathways, are crucial for fibroblast migration, proliferation, and survival. To date, studies on mechanotransduction pathways in IPF have discovered several key signaling pathways, including Rho/ROCK and MRTF-A signaling pathways. Inhibiting the Rho/ROCK pathway reduces lung fibroblast differentiation [32] and α-SMA expression [33], while an MRTF-A knockout also leads to reduced α-SMA expression [34]. Meanwhile, integrin inhibitors have emerged as potential drug targets for IPF treatment in recent years. Clinical trials by Ganesh Raghu et al. showed that the integrin inhibitor BG00011 suppressed TGF-β activation and may interrupt fibrogenesis in a Phase II study [35, 36]. An ongoing clinical trial of Pliant’s dual αvβ1/β6 inhibitor showed potential effectiveness in a Phase II IPF treatment trial [37].

Nintedanib has recently been approved for treating IPF patients. Several clinical studies [38, 39] have demonstrated that the tyrosine kinase receptor inhibitor Nintedanib is a secure and proficient treatment for IPF patients by diminishing the FVC decline rate. A study by Yu et al. [15] determined that Nintedanib therapy could impede bleomycin-induced FAK activation, thereby inhibiting bleomycin-induced endothelial mesenchymal transition. We utilized Defactinib, a selective and effective ATP-competitive FAK inhibitor, to establish the significance of FAK activation in cell fibrosis progression. Our in vitro model of fibrosis confirmed that Defactinib could reduce the expression of the fibrotic marker α-SMA and restore the cellular morphology of fibroblast cells. This led us to hypothesize that FAK-related pathways and associated gene expression levels might be useful in predicting IPF prognosis. Our PTK2 gene signature validated this hypothesis and introduced a new gene signature to predict clinical outcomes for IPF patients.

Our GSEA, GSVA, and KEGG analysis data revealed that most signaling pathways enriched in the high-risk group were immune and cancer-related pathways, such as MAPK, JAK-STAT, VEGF, PI3K-AKT. This indicates that the inflammatory response, which is the primary regulator of IPF’s pathological processes, was heightened in the high-risk group. The fibrogenic cytokine TGF-β can induce the transcription of extracellular signal-regulated protein kinase (ERK1/2) target genes, leading to secondary activation of the PI3K-AKT pathway. The PI3K pathway plays a crucial role in the proliferation and differentiation of myofibroblasts induced by TGF-β [40, 41]. PI3K inhibitors can prevent the TGF-β-induced increase in cell proliferation in IPF [41]. The pathway of VEGF (an angiogenesis factor) activation has been implicated in IPF pathogenesis [42]. VEGF inhibitors demonstrated dose-dependent inhibition of TGF-β-induced differentiation in IPF [43]. The genes in our PTK2-associated gene signature are involved in various biological activities strongly linked to the proliferation and differentiation of myofibroblasts in IPF.

Immune cells play a crucial role in the development of IPF, as confirmed by numerous studies. Our findings regarding immune cell infiltration levels show that the estimated proportions of neutrophils and plasma B cells are increased in the high-risk group, aligning with prior research on immune cells in IPF. Gregory et al. [44] discovered that neutrophil elastase, a neutrophil-derived serine proteinase, could promote myofibroblast differentiation in IPF. Additionally, Achaiah et al. [45] suggested that the blood neutrophil ratio was a prognostic indicator of disease progression, with an elevated neutrophil ratio being linked to rapid lung function decline. Regarding plasma cells, Xue et al. [46] demonstrated that abnormalities in plasma B cells were strongly associated with patients exhibiting reduced survival years. Meanwhile, our study found that the estimated proportion of CD4+ T cells was decreased in the high-risk group. CD4+ T cells in the blood may have a protective role in IPF, as identified in a study that showed a decreased percentage of CD4+ CD28+ T cells in PBMCs could reduce transplant-free survival [22].

Our study’s findings indicate that our gene signature serves not only as a statistical tool for predicting IPF prognosis but also offers valuable insights into the underlying pathological processes, immune responses, and signaling pathways; however, this study has limitations. First, our transcriptome analysis from microarray datasets cannot reveal alterations in overall immune status and pathways, thus, limiting our ability to make generalizations about the overall pathway. Second, since the datasets are independent and from different studies, some clinical characteristics may be incomplete, such as lung function data and grading. Third, the protein levels associated with these genes have yet to be established. Future tests using proteomics techniques could uncover these changes. Fourth, the stage of IPF at the time of blood sample collection could impact our prognostic model. Future investigations will be conducted to examine how the stage of IPF influences our blood-borne gene signature. Therefore, these findings should be interpreted with caution.

In summary, our study developed a novel PTK2-associated gene signature with the potential to predict prognosis, serving as a valuable biomarker and therapeutic target for patients with IPF.

### Supplementary Information

**Additional file 1: Table S1.** Genes Associated with PTK2. This supplementary table presents the names of all genes associated with PTK2 and their corresponding node degrees obtained from the STRING database.

**Additional file 2: Figure S1.** Defactinib (FAK inhibitor) reduced fibrotic markers in IMR90 Fibroblast cells. A) Defactinib reduced TGF-β activated fibrotic markers, B) pY397-FAK, and alpha smooth muscle actin (α-SMA), in IMR-90 cells, C) cellular morphology of IMR90.

**Additional file 3: Figure S2.** Defactinib (FAK inhibitor) reduced fibrotic markers in DHLF-IPF cells. A) Defactinib reduced fibrotic markers, B) pY397-FAK, and alpha smooth muscle actin (α-SMA), in DHLF-IPF cells, C) cellular morphology of DHLF-IPF cells.

**Additional file 4: Figure S3.** RAW Image of pY397-FAK. Western blot analysis was conducted for pY397-FAK.

**Additional file 5: Figure S4.** RAW Image of FAK. Western blot analysis was conducted for FAK.

**Additional file 6: Figure S5**. RAW Image of ⍺-SMA. Western blot analysis was conducted for ⍺-SMA.

**Additional file 7: Figure S6.** RAW Image of β-actin. Western blot analysis was conducted for β-actin.

## Data Availability

Data available within the article or its supplementary materials.

## Abbreviations

IPF: Idiopathic pulmonary fibrosis
FVC: Forced vital capacity
HRCT: High-resolution computed tomography
GAP: Gender, age, lung physiology
CPI: Composite physiologic index
TGF-β: Transforming growth factor-beta
ECM: Extracellular matrix
FAK: Focal adhesion kinase
PTK2: Protein tyrosine kinase 2
GEO: Gene Expression Omnibus
PPI: Protein–protein interaction
KEGG: Kyoto Encyclopedia of Genes and Genomes
ROC: Receiver operating characteristic
DEGs: Differentially expressed genes
GSVA: Gene set variation analysis
GSEA: Gene set enrichment analysis
PBMCs: Peripheral blood mononuclear cells
