# Supplementary figures and images for: PTK2-associated gene signature could predict the prognosis of IPF
Source: Respir Res. 2023 Dec 6;24:304. doi: 10.1186/s12931-023-02582-4 (PMC10699084; doi:10.1186/s12931-023-02582-4)

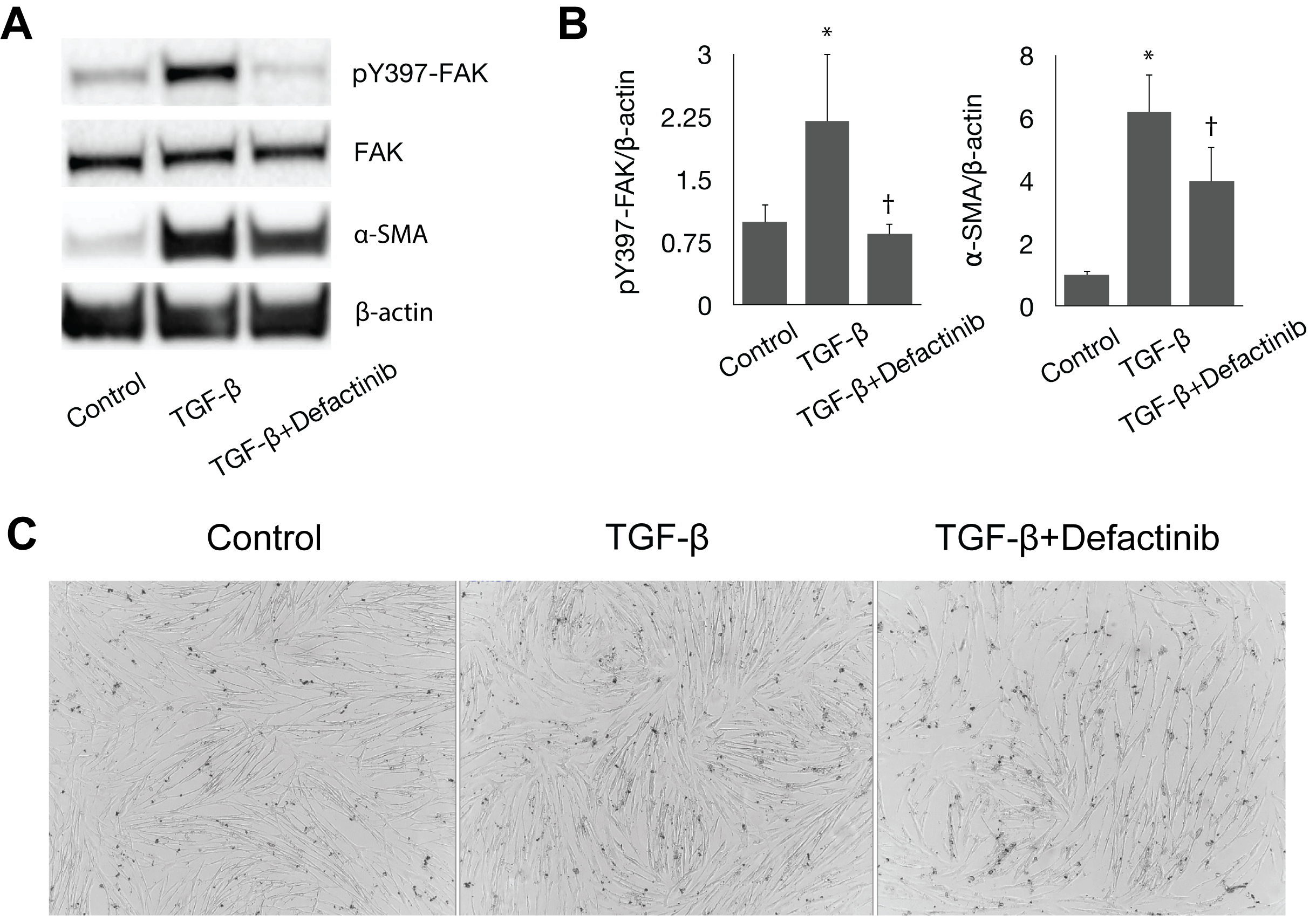

Supplement: Supplementary file 2 — Additional file 2: Figure S1. Defactinib (FAK inhibitor) reduced fibrotic markers in IMR90 Fibroblast cells. A) Defactinib reduced TGF-β activated fibrotic markers, B) pY397-FAK, and alpha smooth muscle actin (α-SMA), in IMR-90 cells, C) cellular morphology of IMR90. [file 12931_2023_2582_MOESM2_ESM.tif]

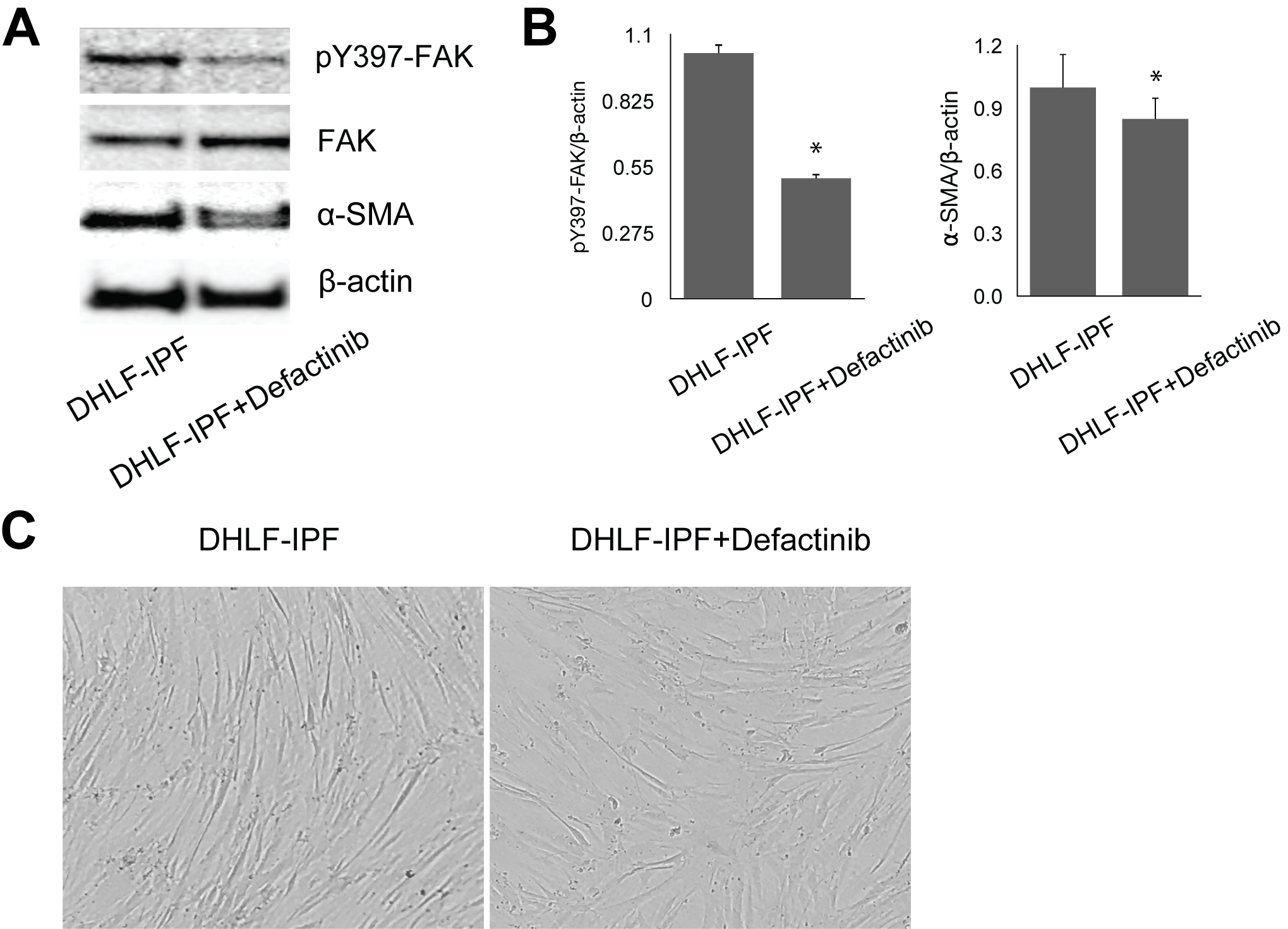

Supplement: Supplementary file 3 — Additional file 3: Figure S2. Defactinib (FAK inhibitor) reduced fibrotic markers in DHLF-IPF cells. A) Defactinib reduced fibrotic markers, B) pY397-FAK, and alpha smooth muscle actin (α-SMA), in DHLF-IPF cells, C) cellular morphology of DHLF-IPF cells. [file 12931_2023_2582_MOESM3_ESM.tif]

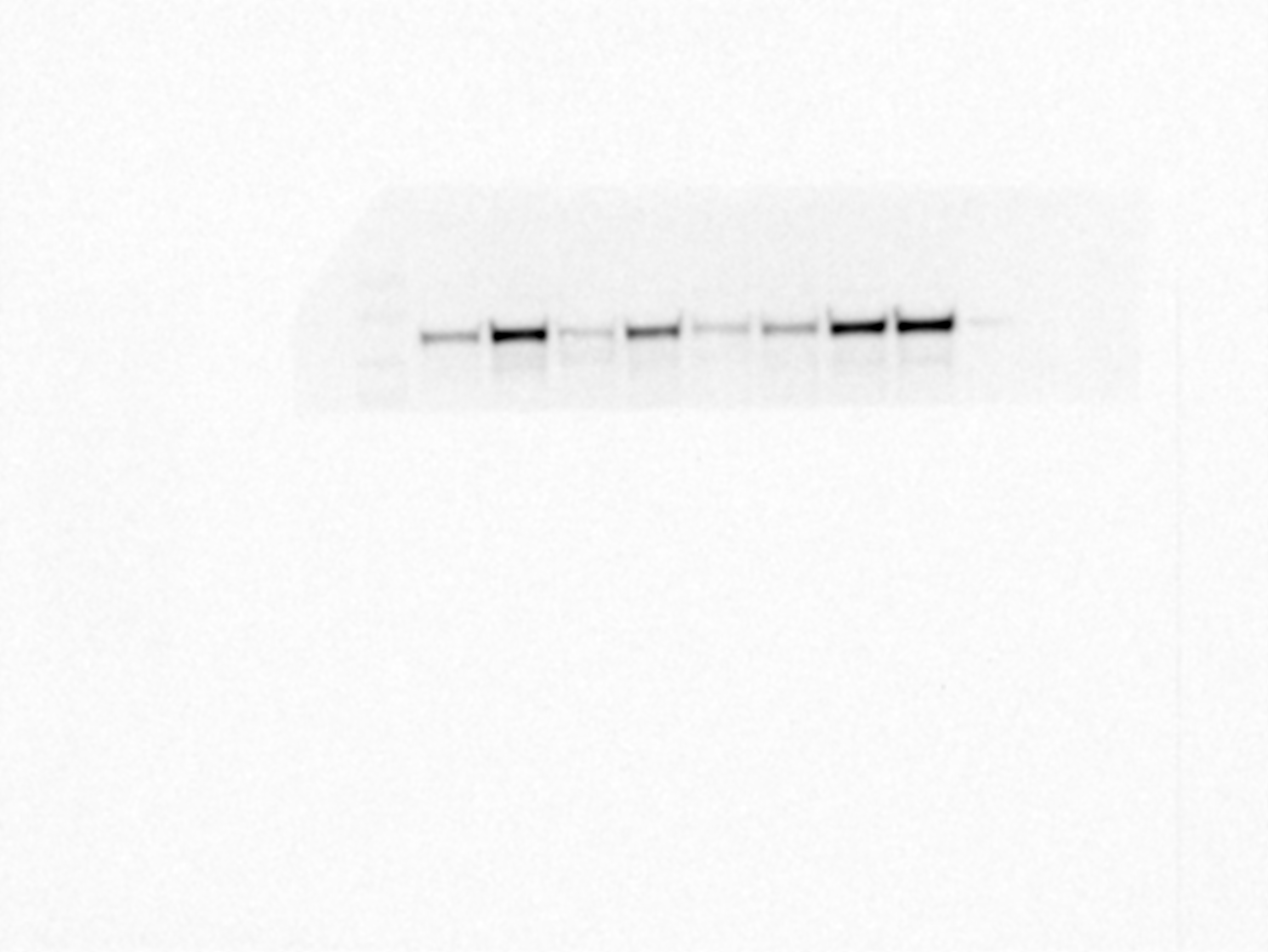

Supplement: Supplementary file 4 — Additional file 4: Figure S3. RAW Image of pY397-FAK. Western blot analysis was conducted for pY397-FAK. [file 12931_2023_2582_MOESM4_ESM.tif]

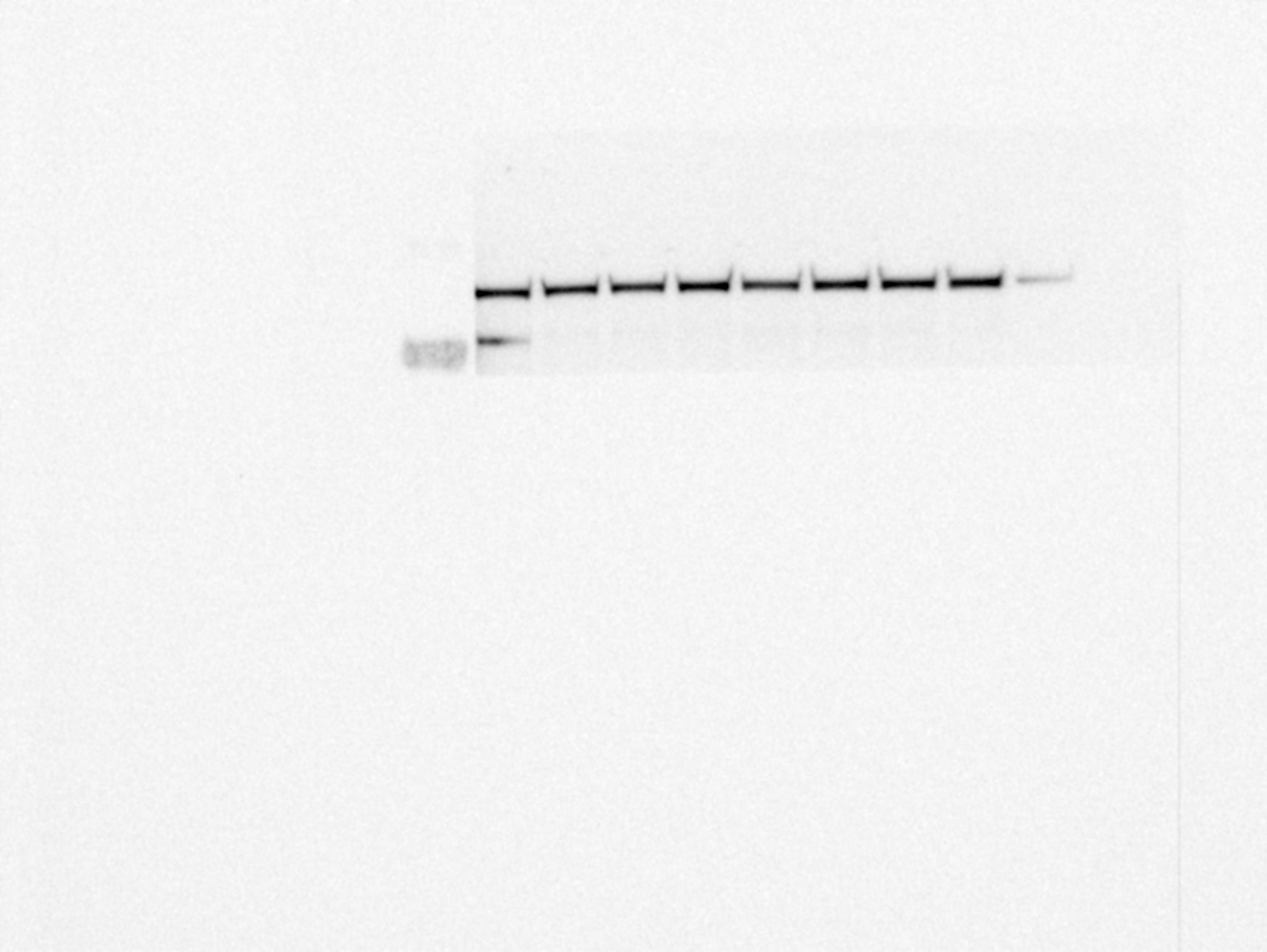

Supplement: Supplementary file 5 — Additional file 5: Figure S4. RAW Image of FAK. Western blot analysis was conducted for FAK. [file 12931_2023_2582_MOESM5_ESM.tif]

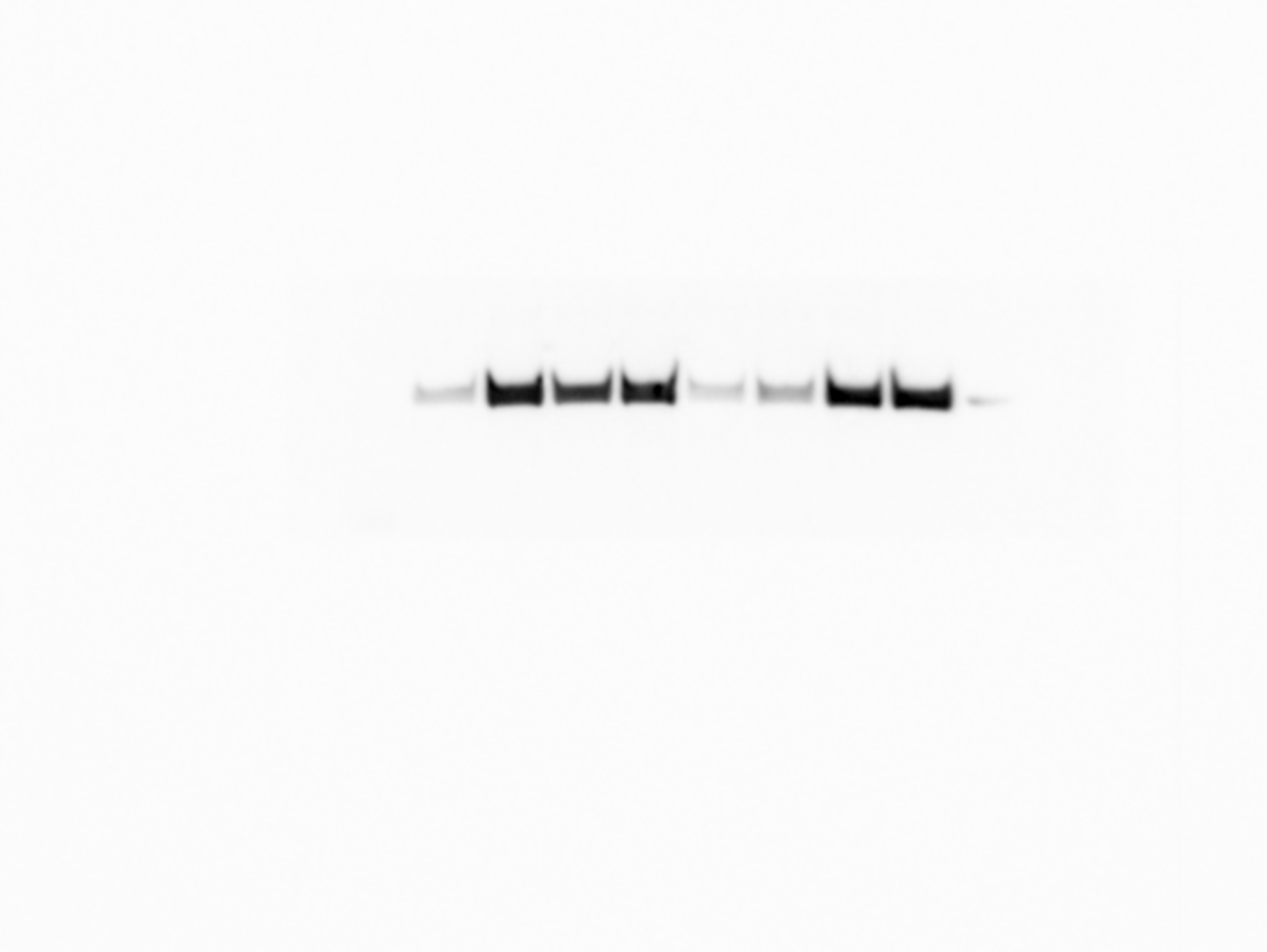

Supplement: Supplementary file 6 — Additional file 6: Figure S5. RAW Image of ⍺-SMA. Western blot analysis was conducted for ⍺-SMA. [file 12931_2023_2582_MOESM6_ESM.tif]

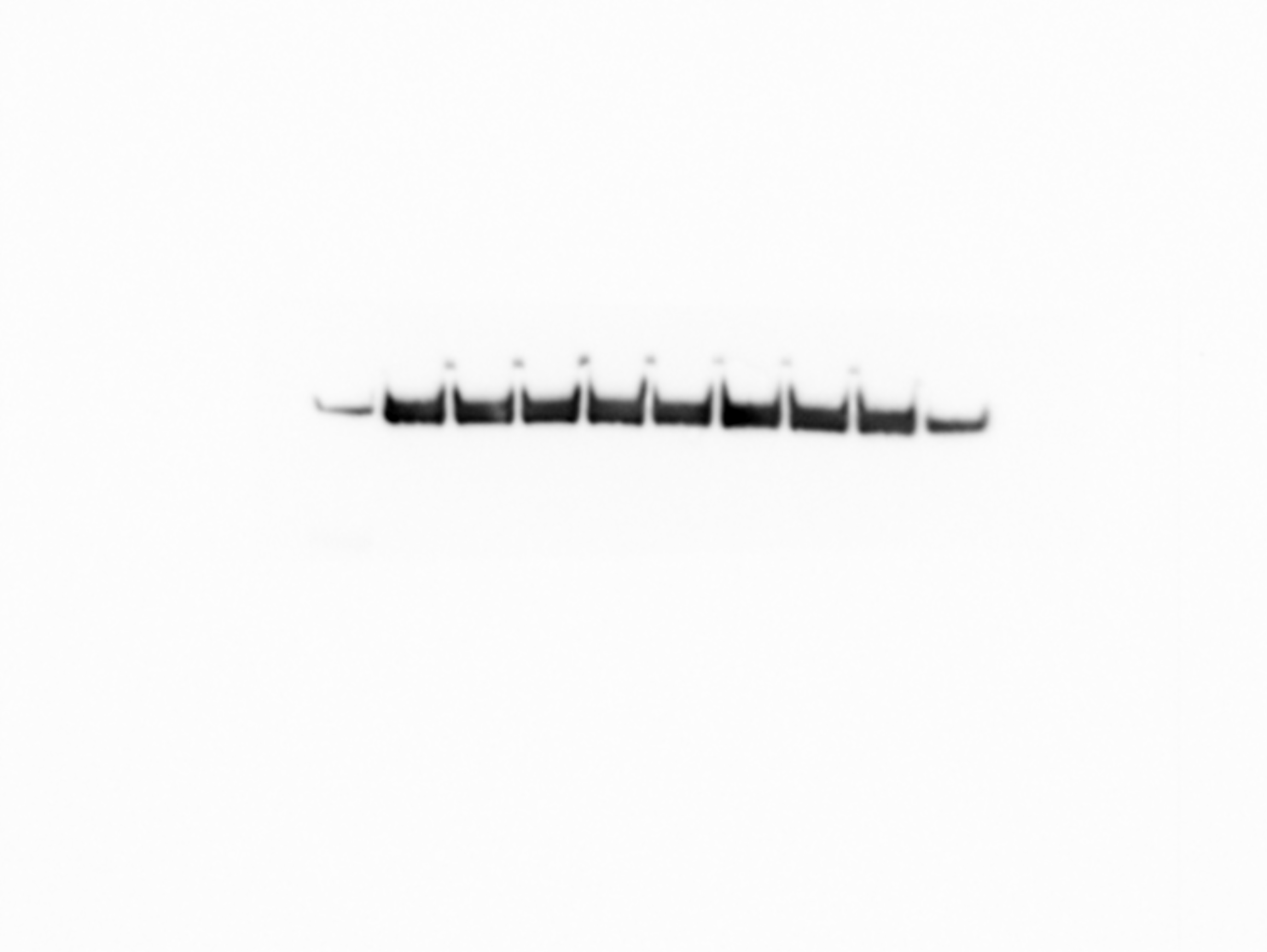

Supplement: Supplementary file 7 — Additional file 7: Figure S6. RAW Image of β-actin. Western blot analysis was conducted for β-actin. [file 12931_2023_2582_MOESM7_ESM.tif]
